# Deletion of IL-4Rα signaling on B cells limits hyperresponsiveness depending on antigen load

**DOI:** 10.1016/j.jaci.2020.12.635

**Published:** 2021-07

**Authors:** Sabelo Hadebe, Jermaine Khumalo, Sandisiwe Mangali, Nontobeko Mthembu, Hlumani Ndlovu, Martyna Scibiorek, Amkele Ngomti, Frank Kirstein, Frank Brombacher

**Affiliations:** a Division of Immunology, and South African Medical Research Council Immunology of Infectious Diseases, Department of Pathology, Faculty of Health Sciences, University of Cape Town, Cape Town, South Africa; b International Centre for Genetic Engineering and Biotechnology (ICGEB) and Institute of Infectious Diseases and Molecular Medicine (IDM), Division of Immunology, Health Science Faculty, University of Cape Town, Cape Town, South Africa; c Division of Chemical and Systems Biology, Department of Integrative Biomedical Sciences, Faculty of Health Sciences, Institute of Infectious Diseases and Molecular Medicine, University of Cape Town, Cape Town, South Africa; d Wellcome Centre for Infectious Diseases Research in Africa (CIDRI-Africa), Institute of Infectious Diseases and Molecular Medicine (IDM), Faculty of Health Sciences, University of Cape Town, Cape Town, South Africa

**Keywords:** IL-4Rα, T_H_2 cells, B cells, germinal center, T follicular helper cell, B effector 2 cells, AHR, Airway hyperresponsiveness, APC, Allophycocyanin, Be2, B effector 2, GC, Germinal center, HDM, House dust mite, IL-4Rα, IL-4 receptor alpha, mLN, Mediastinal lymph node, T_FH_, T follicular helper

## Abstract

**Background:**

B cells play an important role in allergies through secretion of IgE. IL-4 receptor α (IL-4Rα) is key in allergic asthma and regulates type 2 cytokine production, IgE secretion, and airway hyperresponsiveness. IL-4 activation of B cells is essential for class switching and contributes to the induction of B effector 2 (Be2) cells. The role of Be2 cells and signaling via IL-4Rα in B cells is not clearly defined.

**Objective:**

We sought to find out whether IL-4Rα–responsive B cells or Be2 function was essential in experimental allergic asthma.

**Methods:**

Mice lacking IL-4Rα on B cells (mb1^cre^IL-4Rα^−/lox^) or littermate controls (IL-4Rα^−/lox^) and mice lacking IL-4 or IL-4/IL-13 on B cells were sensitized and challenged with high-dose house dust mite (>10 μg) or with low-dose house dust mite (<3 μg). We also adoptively transferred naive IL-4Rα^−/lox^ or IL-4Rα^−/−^ B cells into μMT^−/−^ mice a day before sensitization or a day before challenge. We analyzed lung inflammation, cellular infiltrate, and airway hyperresponsiveness.

**Results:**

We found that IL-4Rα signaling on B cells was important for optimal T_H_2 allergic immune responses mainly when the load of antigen is limited. IL-4Rα signaling on B cells was essential for germinal centers and in the effector phase of allergic responses. Be2 cells were essential in airway hyperresponsiveness, but not in other parameters.

**Conclusions:**

IL-4Rα signaling on B cells is deleterious in allergic asthma because it is required for optimal T_H_2 responses, Be2 function, germinal center formation, and T follicular helper cells, especially when the load of the antigen is limiting.

## Graphical abstract

**Figure:**
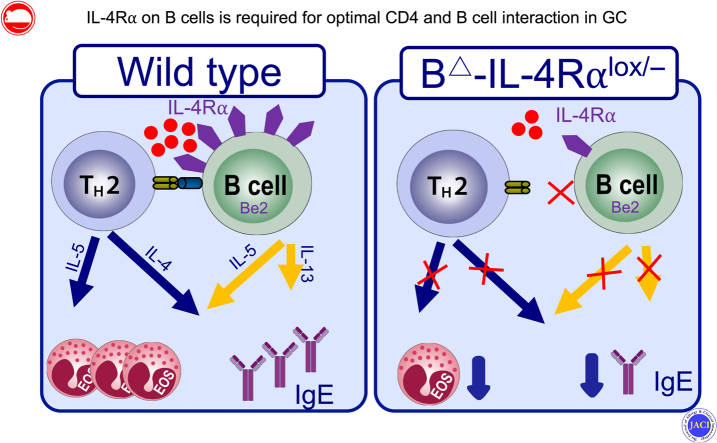

Asthma is a chronic debilitating disease affecting more than 300 million people worldwide, with at least 250,000 people dying from complications associated with the disease.^1^ The immune response to the disease is characterized by T_H_2 immune cells such as eosinophils and type 2 cytokines IL-4, IL-5, and IL-13 and B cells secreting IgE.^2^^,^^3^ Secreted IgE binds to high-affinity receptors FcεR on the surfaces of mast cells and basophils, resulting in activation and degranulation of these cells and release of histamines, proteases, and membrane phospholipids such as leukotrienes and prostaglandins.^4^^,^^5^ Priming of long-lived type 2 memory T cells is attributed to dendritic cells,^6^^,^^7^ with earlier studies demonstrating a minimal contribution from B cells, despite their ability to present antigens to T cells.^8^^,^^9^

The role of B cells in experimental allergic asthma is contradictory; earlier studies using ovalbumin as a model antigen showed a redundant role for B cells in allergic asthma.^10^, ^11^, ^12^ Mice deficient of B cells (μMT^−/−^) developed similar airway hyperresponsiveness (AHR), eosinophilia, and T_H_2 airway responses when sensitized and challenged with ovalbumin.^10^, ^11^, ^12^ More recent evidence using a clinically relevant allergen, house dust mite (HDM), suggests an essential function of B cells in allergic asthma.^13^, ^14^, ^15^ However, despite empirical evidence demonstrating a key role played by B cells in allergic asthma, caveats and contradictions in literature still exist and require further clarification. Some studies have suggested that B cells are either not important at all^9^ or are essential only in priming of CD4 T cells and induction of T follicular helper (T_FH_) cells during the sensitization stage, but play no part during challenge stages of HDM-induced asthma.^13^ Other studies have shown that B cells are essential in both priming of CD4 T cells^15^ and effector stages of HDM-induced allergic asthma.^14^ Furthermore, the load of antigen seems to be critical in the involvement of B cells in HDM-induced allergic asthma.^14^ At high doses of HDM antigen, B cells play minimal role in antigen uptake, processing, and presentation to CD4 T cells, whereas at low doses of HDM antigen, B cells have more access to antigen and can uptake, process, and present antigen to T cells, playing an essential part in the development of T_FH_ cells.^14^ Interestingly, the inability of B cells to present antigen at the sensitization stage leads to T_H_1 and T_H_17 airway responses and not T_H_2 responses.^15^

IL-4 receptor alpha (IL-4Rα) is central in T_H_2 allergic airway asthma^16^, ^17^, ^18^ and other type 2 diseases.^19^^,^^20^ In allergic asthma, we and others have shown temporal^21^ and cell-specific requirement of IL-4Rα in dendritic cells,^22^ T cells,^23^ and epithelial cells^24^ and also the redundant role of this IL-4/IL-13 signaling receptor in macrophages^25^ and airway smooth muscle cells.^26^^,^^27^

We have recently shown that early IL-4 production by B cells influences type 2 CD4 T-cell differentiation in lymph nodes, which leads to protective type 2 responses against certain parasitic infections.^28^, ^29^, ^30^ Given the complexity around the importance of B cells in allergic asthma, we set to investigate the role of IL-4Rα signaling, specifically in B cells, during HDM-induced allergic asthma.

We challenged mice with high-dose or low-dose HDM to assess whether antigen load matters in the requirement of IL-4Rα–responsive B cells in allergic asthma. We found that although mice lacking IL-4Rα on B cells (mb1^cre^IL-4Rα^−/lox^) had reduced AHR at high-dose HDM, in other parameters, they were comparable to littermate control mice. This was contrary to what we observed at low-dose HDM, where mb1^cre^IL-4Rα^−/lox^ mice had reduced AHR, type 2 responses, eosinophilia, T_FH_ cells, and ability to produce T_H_2 cytokines. By adoptively transferring naive IL-4Rα–deficient B cells into μMT^−/−^ mice sensitized to low-dose HDM, we demonstrated the importance of IL-4Rα signaling on B cells at both sensitization and effector stages. Interestingly, lack of IL-4 or IL-4/IL-13 production by B cells resulted in reduced AHR, which suggested a key contribution in this parameter, but less so in airway inflammation or antibody production.

Here, we show an essential role for IL-4Rα–responsive B cells in optimal type 2 allergic airway inflammation, especially when the load of HDM is limited.

## Methods

### Mice

To generate mice deficient of IL-4Rα only on B cells (mb1^cre^IL-4Rα^−/lox^), we intercrossed homozygous mb1^cre^ mice^31^ with IL-4Rα^−/−^^32^ on Balb/c background. We then further mated mb1^cre^ IL-4Rα^−/−^ mice with homozygous IL-4Rα^lox/lox^ mice^19^ to generate hemizygous mb1^cre^IL-4Rα^−/lox^^33^ mice, which were backcrossed up to 10 generations in Balb/c background. Hemizygous littermates (IL-4Rα^−/lox^) expressing single functional IL-4Rα allele was used as a wild-type control in all experiments. Mice were housed in independently ventilated cages under specific pathogen-free conditions at the University of Cape Town Animal Facility. All mice were used at age 8 to 10 weeks, and animal procedures were performed according to strict recommendation by the South African Veterinary Council and were approved by the University of Cape Town Animal Ethics Committee (reference no. 018/013).

### HDM-induced allergic airway disease

A high-dose and a low-dose treatment schedule was used to induce symptoms of allergic asthma in mice.^14^ Mice were anesthetized with ketamine 80 mg/kg (Anaket-V; Centaur Labs, Johannesburg, South Africa) and xylazine 16 mg/kg (Rompun; Bayer, Isando, South Africa). For the high-dose schedule, mice were sensitized intratracheally on day 0 with 100 μg of HDM (Stellergens Greer Laboratories, Lenoir, NC) and intranasally challenged with 10 μg HDM on days 8, 9, 10, 11, and 12. For low-dose treatment, mice were sensitized with 1 μg and challenged with 3 μg of HDM. AHR was measured on day 15. After the procedure, mice were euthanized and tissue samples were collected for analysis.

### Adoptive transfer

Spleens were collected from naive IL-4Rα^−/lox^, mb1^cre^IL-4Rα^−/lox^, IL-4^−/−^, or IL-4/IL-13^−/−^ mice and passed through a 40-μm strainer to obtain single-cell suspensions. Cells were stained with Flourescein isothiocyanate (FITC)-B220 and allophycocyanin (APC)-CD19 for 30 minutes at 4^o^C. A dead cell exclusion dye (7AAD) was added before sorting on BD FACS Aria I to at least 96% purity. A total of 2 × 10^6^ to 5 × 10^6^ cells were adoptively transferred intravenously into μMT^−/−^ recipient mice a day before HDM sensitization. In other experiments, sorted B cells were adoptively transferred intravenously into low-dose HDM-sensitized mice a day before challenge with low-dose HDM.

### Airway hyperresponsiveness

Airway resistance and elastance of the whole respiratory system (airways, lung chest wall) after intranasal challenge were determined by forced oscillation measurements as described previously^25^ with the Flexivent system (SCIREQ, Montreal, Canada) by using the single-compartment (‘‘snapshot’’) perturbation. Measurements were carried out on mice with increasing doses (0, 5, 10, 20, and 40 mg/mL) of acetyl-β-methylcholine (methacholine, Sigma-Aldrich, Aston Manor, South Africa) treatment. Differences in the dose-response curves were analyzed by repeated-measures 2-way ANOVA with the Bonferroni posttest. Only mice with acceptable measurements for all doses (coefficient of determination >0.90) were included in the analysis.

### Flow cytometry

Bronchoalveolar lavage fluid cells were obtained as previously described.^26^ Single-cell suspensions were prepared from lymph nodes in RPMI media (Gibco, Paisley, United Kingdom) by passing them through a 100-μm strainer. To obtain single-cell suspensions from lung tissues, a left lobe was digested for 1 hour at 37^o^C in RPMI media containing 13 mg/mL DNase I (Roche, Randburg, South Africa) and 50 U/mL collagenase IV (Gibco, Waltham, Mass) and passed through a 70-μm strainer. Antibodies used in these experiments included the following: phycoerythrobilin-conjugated anti–Siglec-F (clone E50-2440), anti-CD124 (IL-4Rα, clone M-1), anti–IL-5 (clone TRFK5), anti-CD44 (clone KM114), FITC-conjugated anti–Gr-1 (clone RB6-8C5), CD45 (clone 30-F11), IL-4 (clone 11B11), PerCP Cy5.5–conjugated anti-Ly6C (clone AL-21), anti–IL-17 (clone TC11-18H10), APC-conjugated anti-CD11c (clone HL3), anti-FoxP3 (clone MF23), V450-conjugated anti-CD11b (clone M1/70), anti-CD62L (clone MEL-14), anti-IgG_1_ (clone A110-1), AlexaFlour 700–conjugated anti-CD3ε (clone 145-2C11), anti–IFN-γ, V500–anti-CD4 (clone RM4-5) and anti-B220 (clone RA3-6B2), APC-Cy7–conjugated anti-CD19 (clone 1D3) and anti-CD8 (clone 53-6.7), BV786-conjugated anti-IgE (clone R35-72) and anti–IL-33R (ST2) (clone U29-93), and biotin-CD25 (clone, 7D4), which were purchased from BD Pharmingen (San Diego, Calif), phycoerythrobilin-cynanine7 anti-F4/80 (clone BM8), anti–IL-13 (clone eBio13A), AlexaFlouro 700–conjugated anti–MHC II (clone M5/114), APC-conjugated anti–IL-21 (clone FFA21), and live/dead Fixable Yellow stain (Qdot605 dead cell exclusion dye), which were purchased from eBiosciences. Biotin-labeled antibodies were detected by Texas Red–conjugated phycoerythrobilin (BD Biosciences, San Diego, Calif). For staining, cells (1 × 10^6^) were stained and washed in PBS, 3% FCS FACS buffer. For intracellular cytokine staining, cells were restimulated with phorbal 12-myristate 13-acetate (Sigma-Aldrich) (50 ng/mL), ionomycin (Sigma-Aldrich) (250 ng/mL), and monensin (Sigma-Aldrich) (200 mM in Iscove's Modified Dulbecco's Medium (IMDM)/10% FCS) for 5 hours at 37˚C and then fixed in 2% PFA and permeabilized with Foxp3 transcriptional factor staining buffer kit (eBioscience) before intracellular staining with appropriate cytokine antibodies and acquisition through LSR Fortessa machine (BD Immunocytometry System, San Jose, Calif) and data were analyzed using FlowJo software (Treestar, Ashland, Ore).

### Histology

Left upper-lung lobes was fixed in 4% formaldehyde/PBS and embedded in paraffin. Tissue sections were stained with periodic acid-Schiff for mucus secretion and hematoxylin and eosin stain for inflammation. Slides were scanned at 20× magnification on the virtual slide VS120 microscope (Olympus, Hamburg, Germany). Downstream processing of images was done through Image J (FIJI) for image extraction at series 15, and Ilastik software was used for mucus area quantification on whole-lung sections. Data shown are from 1 experiment from at least 3 independent experiments (n = 5-7 mice per experiment).

### Antibody and cytokine ELISAs

Antibody ELISAs were carried out as previously described^26^ using 5 μg/mL HDM to coat for specific IgGs. Total IgE in serum was measured using antimouse IgE (BD Biosciences, 553413) to coat, mouse IgE (κ, anti-TNP, BD Biosciences, 557079) as standard, and biotin antimouse IgE (BD Biosciences, 553419) as secondary antibody.

For *in vitro* cytokine production analysis, single-cell suspensions were prepared from mediastinal lymph nodes (mLNs) of HDM-treated and littermate control mice. Cells (2 × 10^5^ cells, in 200 μL) were incubated for 5 days in IMDM/10% FCS (Delta Bioproducts, Kempton Park, South Africa) in 96-well plates. Cells were stimulated with either HDM (30 μg/mL) or anti-CD3 (10 μg/mL) and supernatants were collected after a 5-day incubation period. Concentrations of IL-4, IL-5 (BD Biosciences), and IL-13 (R&D Systems, Minneapolis, Minn) were measured using ELISAs according to the manufacturer’s protocol.

### Statistical analysis

*P* values were calculated in GraphPad Prism 6 (GraphPad Software, Inc, San Diego, Calif) by using nonparametric Mann-Whitney Student *t* test or 2-way ANOVA with Bonferroni posttest for multiple comparisons, and results are presented as SEM or mean of SD. Differences were considered significant if *P* was less than .05.

## Results

### IL-4Rα–responsive B cells are not essential in high-dose HDM-induced allergic asthma

**Fig 1:**
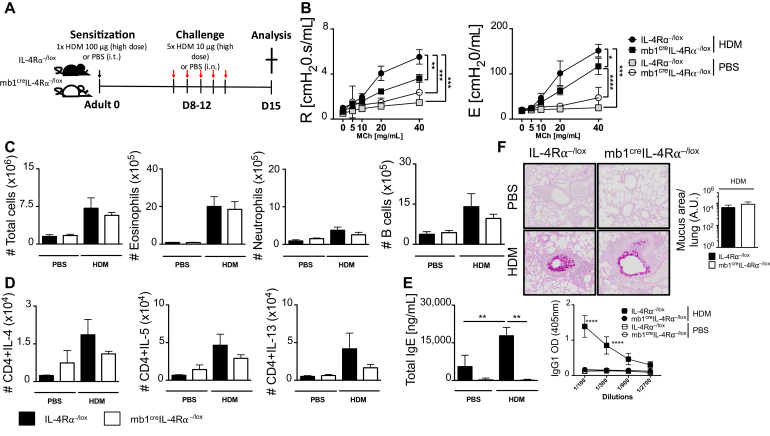
IL-4Rα–responsive B cells regulate AHR and IgE production during high-dose HDM exposure, but have little impact on airway inflammation and T_H_2 responses. **A**, Schematic diagram showing sensitization and challenge protocol where mice (mb1^cre^IL-4Rα^−/lox^) and wild-type littermate control (IL-4Rα^−/lox^) were sensitized with HDM 100 μg intratracheally on day 0 and challenged with HDM 10 μg on days 8 to 12. Analysis was done on day 15. **B,** Airway resistance and elastance were measured with increasing doses of acetyl methacholine (0-40 mg/mL). **C,** Total lung cell numbers, eosinophil numbers, neutrophil numbers, and B-cell numbers were stained and analyzed by flow cytometry and enumerated from % of live cells. **D,** Number of lung CD4 T cells producing IL-4, IL-5, and IL-13 after 5-hour stimulation with PMA/ionomycin in the presence of monensin. **E**, Total serum IgE and HDM-specific IgG_1_ titers measured by ELISA. **F,** Histology analyses of lung sections (magnification ×20), stained with periodic acid-Schiff. *A.U.*, Arbitrary units; *i.n.*, intranasal; *i.t.*, intratrachael; *OD*, optical density; *PMA*, phorbal 12-myristate 13-acetate. Shown are means ± SDs from 1 representative experiment of 2 (n = 4-6). Significant differences between groups were analyzed by Student *t* test (Mann-Whitney) (Fig 1, *C*, *D*, and *F*) or by 2-way ANOVA with Benforroni posttest (Fig 1, *B* and *E*) and are described as ∗*P* < .05, ∗∗*P* < .01, ∗∗∗*P* < .001, ∗∗∗∗*P* < .0001.

**Fig E1:**
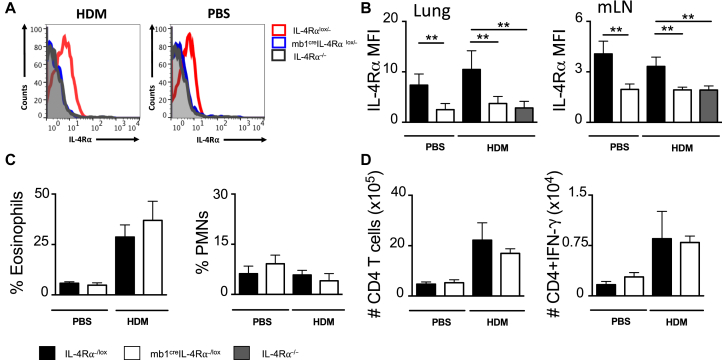
Characterization of IL-4Rα expression on B cells. **A,** Representative histogram plots of IL-4Rα expression on B cells in mLNs. **B,** Quantification of IL-4Rα expression on B cells in mLNs and lungs represented as MFI. **C,** Frequencies of eosinophils (live^+^CD11c^low^ CD11b^high^ Ly6G^low^ SiglecF^hi^) and neutrophils (live^+^CD11c^low^CD11b^high^Ly6G^high^) analyzed by flow cytometry (part of Fig 1). **D,** Total number of lung CD4 T cells and CD4 T cells producing IFN-γ after 5-hour stimulation with PMA/ionomycin in the presence of monensin in mb1^cre^IL-4Rα^−/lox^ and littermate control IL-4Rα^−/lox^ mice. Shown are means ± SDs from 1 representative experiment of 3 or more (n = 6-7). Significant differences between groups were analyzed by Student *t* test (Mann-Whitney) and are described as ∗∗*P* < .01. *MFI*, Median fluorescent intensity; *PMA*, phorbal 12-myristate 13-acetate; *PMN*, polymorphonuclear.

The role of B cells in asthma is controversial,^9^^,^^14^ and recent evidence suggested that the load of antigen is crucial in influencing the role of B cells.^14^ We used a standard high dose of 100 μg HDM to sensitize mice at day 0 and challenged with a reduced dose of 10 μg on days 8 to 12^34^^,^^35^ (Fig 1, *A*). First, we showed that at both steady state and during HDM challenge, there was reduced IL-4Rα expression in both lung and mLNs in mice lacking IL-4Rα on B cells (mb1^cre^IL-4Rα^−/lox^) when compared with littermate (IL-4Rα^−/lox^) control mice or IL-4Rα–deficient mice (see Fig E1, *A* and *B*, in this article’s Online Repository at www.jacionline.org). We found that mice lacking IL-4Rα on B cells had a moderately reduced airway resistance and elastance when compared with littermate mice sensitized and challenged with high-dose HDM (Fig 1, *B*).

We then measured cellular infiltrates within the lung tissue after HDM challenge and observed a comparable increase in total cellular infiltration, which was mainly represented by eosinophils in both mb1^cre^IL-4Rα^−/lox^ and littermate mice challenged with HDM (Fig 1, *C*, and Fig E1, *C*). We then measured type 2 cytokines produced by CD4 T cells in the lung after stimulation with phorbal 12-myristate 13-acetate/ionomycin for 5 hours. We observed increased but comparable levels of CD4 T cells producing IL-4, IL-5, and IL-13 in both mb1^cre^IL-4Rα^−/lox^ and littermate mice challenged with high-dose HDM (Fig 1, *D*). Levels of CD4 T cells producing IL-4, IL-5, and IL-13 were low in mb1^cre^IL-4Rα^−/lox^ and littermate control IL-4Rα^−/lox^ mice challenged with PBS when compared with high-dose HDM-challenged mice of the same genotype (Fig 1, *D*). We also observed no differences in CD4 T-cell numbers or IFN-γ–producing CD4 T cells in all mutants challenged with high-dose HDM (Fig E1, *D*). We observed significantly higher total IgE production and HDM-specific IgG_1_ titers in IL-4Rα^−/lox^ mice when compared with mb1^cre^IL-4Rα^−/lox^ mice challenged with high-dose HDM (Fig 1, *E*). We observed no differences in mucus production and inflammation between mb1^cre^IL-4Rα^−/lox^ and IL-4Rα^−/lox^ mice (Fig 1, *F*). Overall, these results demonstrated that IL-4Rα on B cells plays a minimal role in the development of allergic asthma after a challenge with high-dose HDM.

### IL-4Rα–responsive B cells play an essential role in low-dose HDM-induced allergic asthma

**Fig 2:**
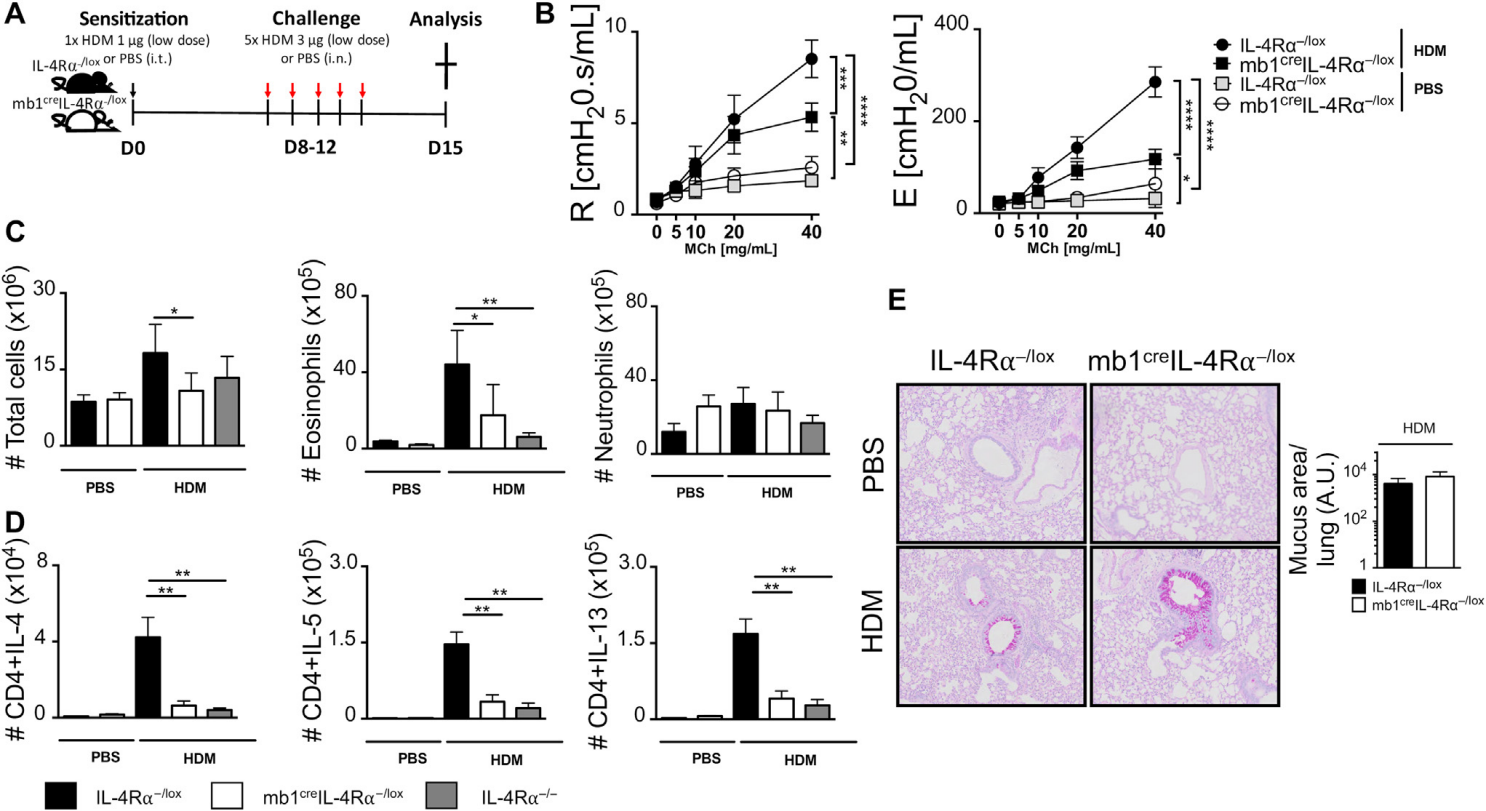
IL-4Rα–responsive B cells are essential in optimal T_H_2 immune responses during low-dose HDM exposure. **A**, Schematic diagram showing sensitization and challenge protocol where mice (mb1^cre^IL-4Rα^−/lox^) and wild-type littermate control (IL-4Rα^−/lox^) were sensitized with HDM 1 μg intratracheally on day 0 and challenged with HDM 3 μg on days 8 to 12. Analysis was done on day 15. **B,** Airway resistance and elastance were measured with increasing doses of acetyl methacholine (0-40 mg/mL). **C,** Total lung cell numbers, eosinophil numbers, and neutrophil numbers were stained and analyzed by flow cytometry and enumerated from % of live cells. **D,** Number of lung CD4 T cells producing IL-4, IL-5, and IL-13 after 5-hour stimulation with PMA/ionomycin in the presence of monensin. Representative FACS plots are shown in Fig E2. **E**, Histology analyses of lung sections (magnification ×20), stained with periodic acid-Schiff. *A.U.*, Arbitrary units; *i.n.*, intranasal; *i.t.*, intratrachael; *PMA*, phorbal 12-myristate 13-acetate. Shown are means ± SDs from 1 representative experiment of 3 (n = 6-7). Significant differences between groups were analyzed by Student *t* test (Mann-Whitney) (Fig 2, *C*, *D*, and *E*) or by 2-way ANOVA with Benforroni posttest (Fig 2, *B*) and are described as ∗*P* < .05, ∗∗*P* < .01, ∗∗∗*P* < .001, ∗∗∗∗*P* < .0001.

**Fig E2:**
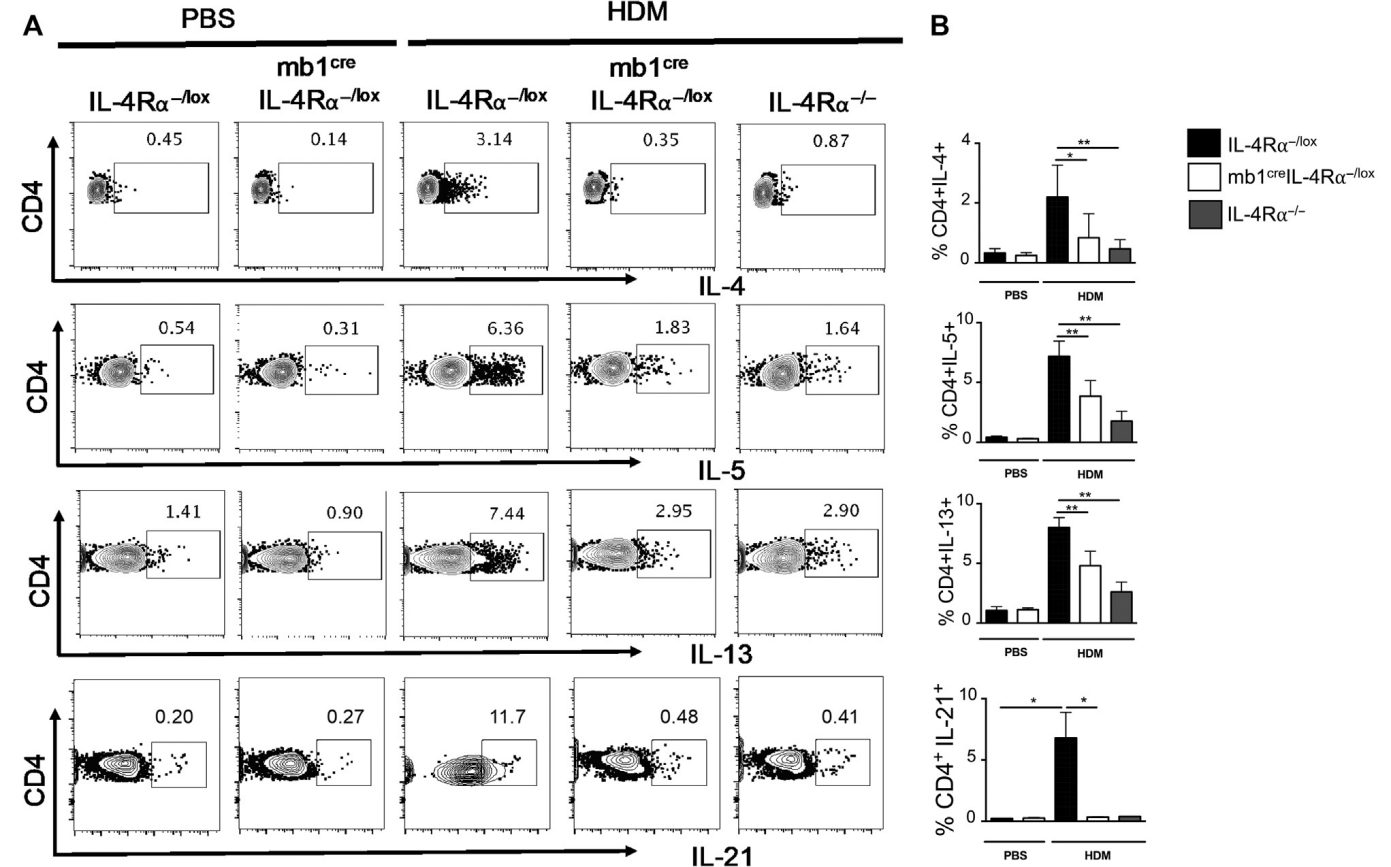
IL-4Rα–responsive B cells are essential in T_H_2 and IL-21 production. Representative flow cytometry plots showing lung CD4 T cells producing IL-4, IL-5, IL-13, and IL-21. Frequencies of CD4^+^cytokine^+^ cells are quantified on the right. This is part of Figs 2 and 3, *D*. Shown are means ± SDs from 1 representative experiment of 3 (n = 4-9). Significant differences between groups were analyzed by Student *t* test (Mann-Whitney) and are described as ∗*P* < .05, ∗∗*P* < .01.

**Fig E3:**
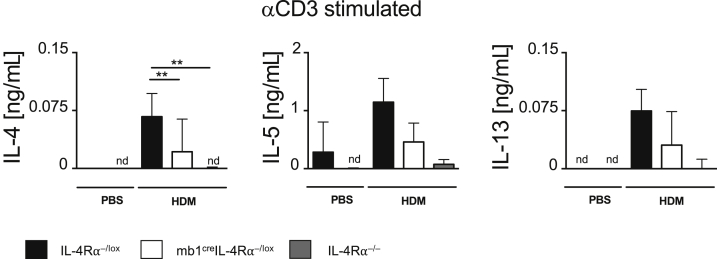
IL-4Rα–responsive B cells are essential in T_H_2 cytokine release after anti-CD3 stimulation. mLNs were stimulated with anti-CD3 (10 μg/mL) for 5 days and supernatants were used to measure levels of IL-4, IL-5, and IL-13. Cytokines were not detected in unstimulated or HDM-stimulated mLNs (30 μg). Shown are means ± SDs from 1 experiment (n = 6-7). Significant differences between groups were analyzed by Student *t* test (Mann-Whitney) and are described as ∗*P* < .01.

B-cell–deficient mice (μMT^−/−^) showed increased eosinophilic airway inflammation when challenged with high- dose HDM, comparable to that observed in wild-type mice, even under chronic challenges.^14^^,^^36^ Titration of HDM below 3 μg reduced the influx of eosinophils, proliferation of Derp-1–specific T cells, and type 2 cytokine production when compared with wild-type mice.^14^ We then used this low-dose HDM sensitization and challenge protocol to assess whether type 2 airway inflammation depended on the dose of inhaled HDM (Fig 2, *A*). We found robust differences in airway resistance and elastance in mb1^cre^IL-4Rα^−/lox^ mice sensitized and challenged with low-dose HDM when compared with littermate IL-4Rα^−/lox^ mice (Fig 2, *B*). Mb1^cre^IL-4Rα^−/lox^ and IL-4Rα^−/lox^ mice challenged with saline had similarly low levels of resistance and elastance compared with HDM-exposed mice (Fig 2, *B*). We then analyzed total lung infiltrate and found a significant increase in total cells and eosinophils in IL-4Rα^−/lox^ littermates compared with mb1^cre^IL-4Rα^−/lox^ or global IL-4Rα–deficient mice challenged with low-dose HDM (Fig 2, *C*). We did not observe any changes in neutrophil numbers when comparing low-dose HDM-challenged mice and control mice that were challenged with saline (Fig 2, *C*). We then analyzed type 2 cytokine production by CD4 T cells in the lung and found a significant increase in percentages and number of CD4 T cells producing IL-4, IL-5, and IL-13 in IL-4Rα^−/lox^ littermate mice when compared with mb1^cre^IL-4Rα^−/lox^ and IL-4Rα^−/−^ mice sensitized and challenged with low-dose HDM (Fig 2, *D*; see Fig E2, *A* and *B*, in this article’s Online Repository at www.jacionline.org). We also found similar trends of reduced T_H_2 cytokine levels in IL-4Rα B-cell–deficient mLNs stimulated for 5 days with anti-CD3 (see Fig E3 in this article’s Online Repository at www.jacionline.org). There were low number of cytokine-producing CD4 T cells in both mb1^cre^IL-4Rα^−/lox^ and littermate control mice sensitized and challenged with saline (Fig 2, *D*). We analyzed lung tissue for signs of inflammation and stained for mucus-producing cells (Fig 2, *E*). We found similar levels of mucus area in both mb1^cre^IL-4Rα^−/lox^ and IL-4Rα^−/lox^ littermate control mice, and there were no detectable mucus-producing cells in control mice challenged with saline (Fig 2, *E*). Overall, these results demonstrate that at low-dose HDM exposure, IL-4Rα on B cells contributes significantly to the development of allergic asthma and T_H_2-type lung inflammation.

### IL-4Rα–responsive B cells are important for accumulation of germinal center B cells and T_FH_ cells in secondary lymphoid tissue at low antigen load

**Fig 3:**
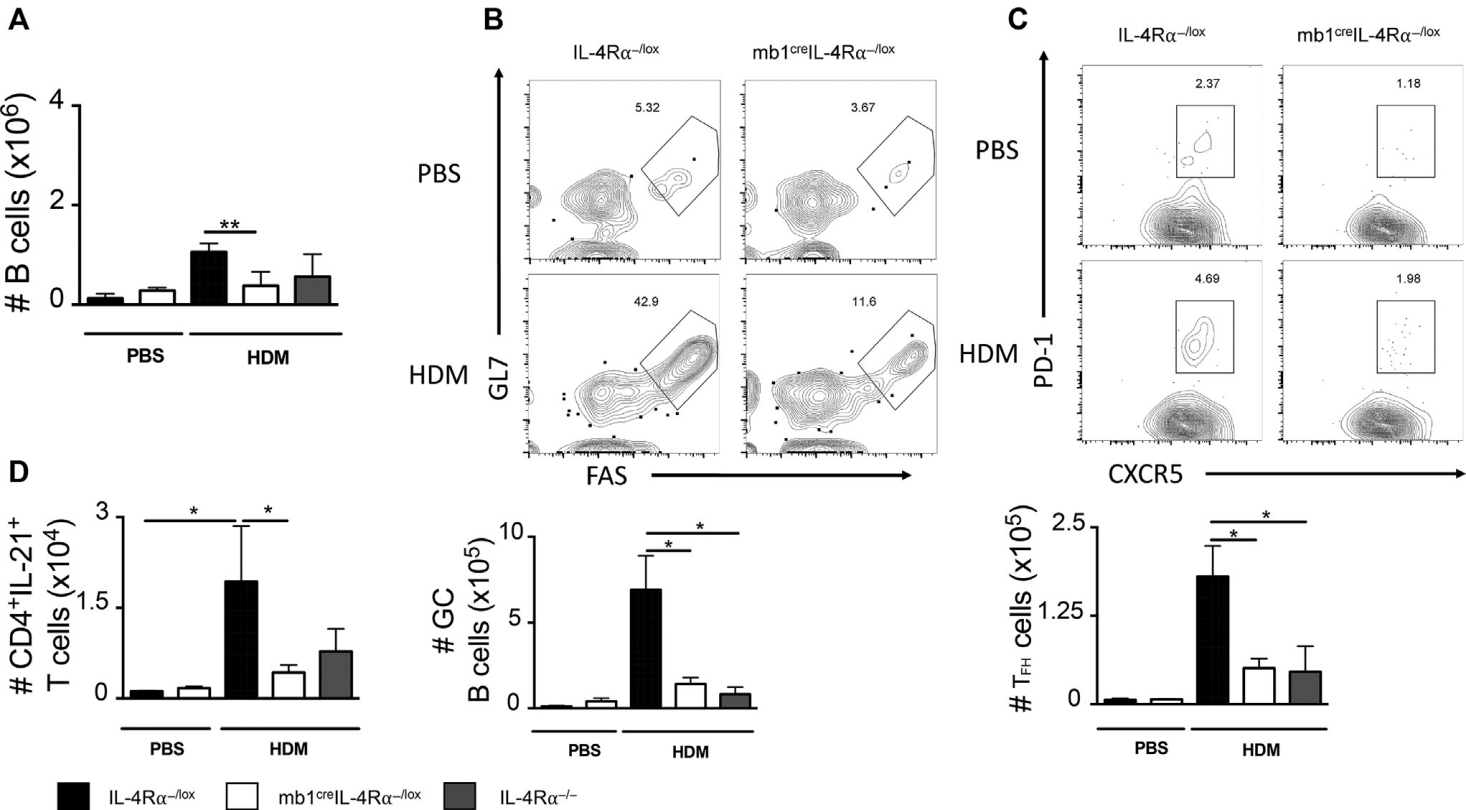
IL-4Rα signaling on B cells is essential for GC formation and T_FH_ cells during low-dose HDM exposure. **A,** Total number of B cells in the mLNs in mb1^cre^IL-4Rα^−/lox^, littermate control (IL-4Rα^−/lox^), and IL-4Rα^−/−^ mice sensitized and challenged as in Fig 2. **B,** Representative flow cytometry plots of GCs and number of GCs (live^+^B220^+^CD19^+^MHCII^+^GL7^+^FAS^+^) in the mLNs in mb1^cre^IL-4Rα^−/lox^ and littermate control IL-4Rα^−/lox^ mice. **C,** Representative flow cytometry plots of T_FH_ cells (live^+^CD3^+^CD4^+^CD44^+^PD-1^+^CXCR5^+^) and number of T_FH_ cells in the mLNs. **D,** Number of lung CD4 T cells producing IL-21 after 5-hour stimulation with PMA/ionomycin in the presence of monensin. Representative FACS plots are shown in Fig E2. *FAS*, *F*S-7-associated surface; *PMA*, phorbal 12-myristate 13-acetate. Shown are means ± SDs from 1 representative experiment of 3 (n = 4-6). Significant differences between groups were analyzed by Student *t* test (Mann-Whitney) and are described as ∗*P* < .05.

**Fig E4:**
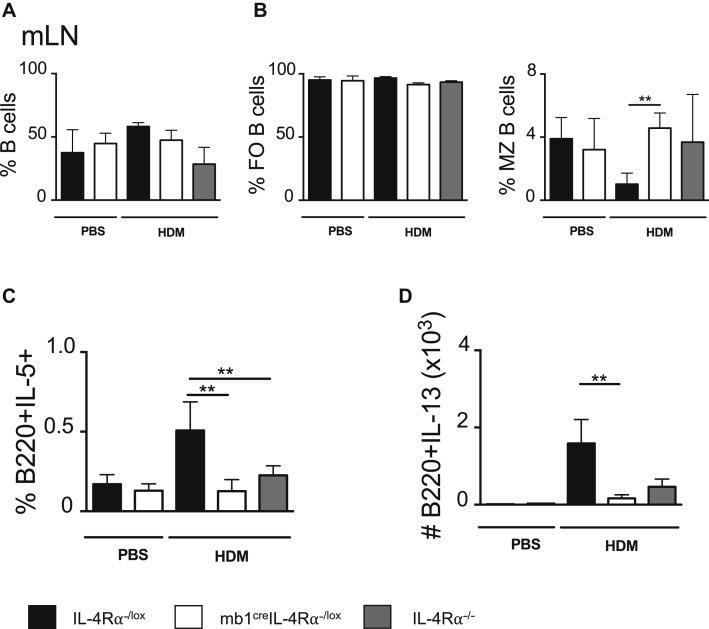
B cells produce other T_H_2-type cytokines. **A,** Frequencies of B cells (live^+^B220^+^CD19^+^MHCII^+^) in mLNs of mb1^cre^IL-4Rα^−/lox^, littermate control IL-4Rα^−/lox^, and IL-4Rα^−/−^ mice. **B,** Frequencies of follicular (FO) B cells (live^+^B220^+^CD19^+^MHCII^+^CD23^+^CD21/CD35^low^) and marginal-zone (MZ) B cells (live^+^B220^+^CD19^+^MHCII^+^CD23^low^CD21/CD35^+^) in mLNs of mb1^cre^IL-4Rα^−/lox^, littermate control IL-4Rα^−/lox^, and IL-4Rα^−/−^ mice. **C,** Frequencies of IL-5–producing B cells (live^+^B220^+^CD19^+^MHCII^+^IL-5^+^) in the lungs of mb1^cre^IL-4Rα^−/lox^, littermate control IL-4Rα^−/lox^, and IL-4Rα^−/−^ mice. **D,** Total number of IL-13–producing B cells (live^+^B220^+^CD19^+^MHCII^+^IL-13^+^) in the lungs of mb1^cre^IL-4Rα^−/lox^, littermate control IL-4Rα^−/lox^, and IL-4Rα^−/−^ mice. Shown are means ± SDs from 1 representative experiment of 3 (n = 6-7). Significant differences between groups were analyzed by Student *t* test (Mann-Whitney) and are described as ∗∗*P* < .01.

B cells have been shown to be important in the development of T_FH_ cells, and these T_FH_ cells acted as precursors for IL-4/IL-13–committed CD4 T cells that migrated to the lung to recruit eosinophils and caused disease.^13^ First, we measured percentages and number of B cells in the mLNs and found frequencies to be intact (see Fig E4, *A*, in this article’s Online Repository at www.jacionline.org). However, total numbers were significantly reduced when comparing mb1^cre^IL-4Rα^−/lox^ to IL-4Rα^−/lox^ littermate mice and global IL-4Rα–deficient mice (Fig 3, *A*). We then compared germinal center (GC) B cells in mLNs and found comparably low levels between mb1^cre^IL-4Rα^−/lox^ and IL-4Rα^−/lox^ littermate control mice (Fig 3, *B*). We observed significantly higher percentages (represented by high expression of GL7 and FAS) and number of GC B cells in IL-4Rα^−/lox^ littermate mice when compared with mb1^cre^IL-4Rα^−/lox^ mice challenged with low-dose HDM (Fig 3, *B*). We did not observe major changes in frequencies of follicular B cells (Fig E4, *B*), but observed significantly increased frequencies of marginal-zone B cells (Fig E4, *B*) in mb1^cre^IL-4Rα^−/lox^ mice when compared with IL-4Rα^−/lox^ littermate mice challenged with low-dose HDM, probably as a compensatory mechanism to increase non-GC antibody production. We then looked for T_FH_ cells of which we know B cells play a crucial role in their development particularly at low-dose HDM. We found significantly reduced frequencies and numbers of T_FH_ cells (represented by high expression of PD-1 and CXCR5) in mLNs of mb1^cre^IL-4Rα^−/lox^ and IL-4Rα^−/−^ when compared with IL-4Rα^−/lox^ littermate controls (Fig 3, *C*). To understand whether these T_FHs_ could be contributing to effector T_H_2 cells in the lung, we analyzed IL-21 intracellular levels produced by CD4 T cells in the lung.^37^ We found significantly reduced frequencies and numbers of IL-21–producing T cells in mb1^cre^IL-4Rα^−/lox^ and IL-4Rα^−/−^ when compared with IL-4Rα^−/lox^ littermate controls (Fig 3, *D*, and Fig E2, *A* and *B*). Our data suggested that IL-4Rα–responsive B cells in secondary lymphoid tissues are important for the accumulation of GC B cells and development of T_FH_ cells, which might be contributing to overall T_H_2 cells.

### IL-4Rα–responsive B cells are required for optimal T_H_2 airway responses and antibody production

**Fig 4:**
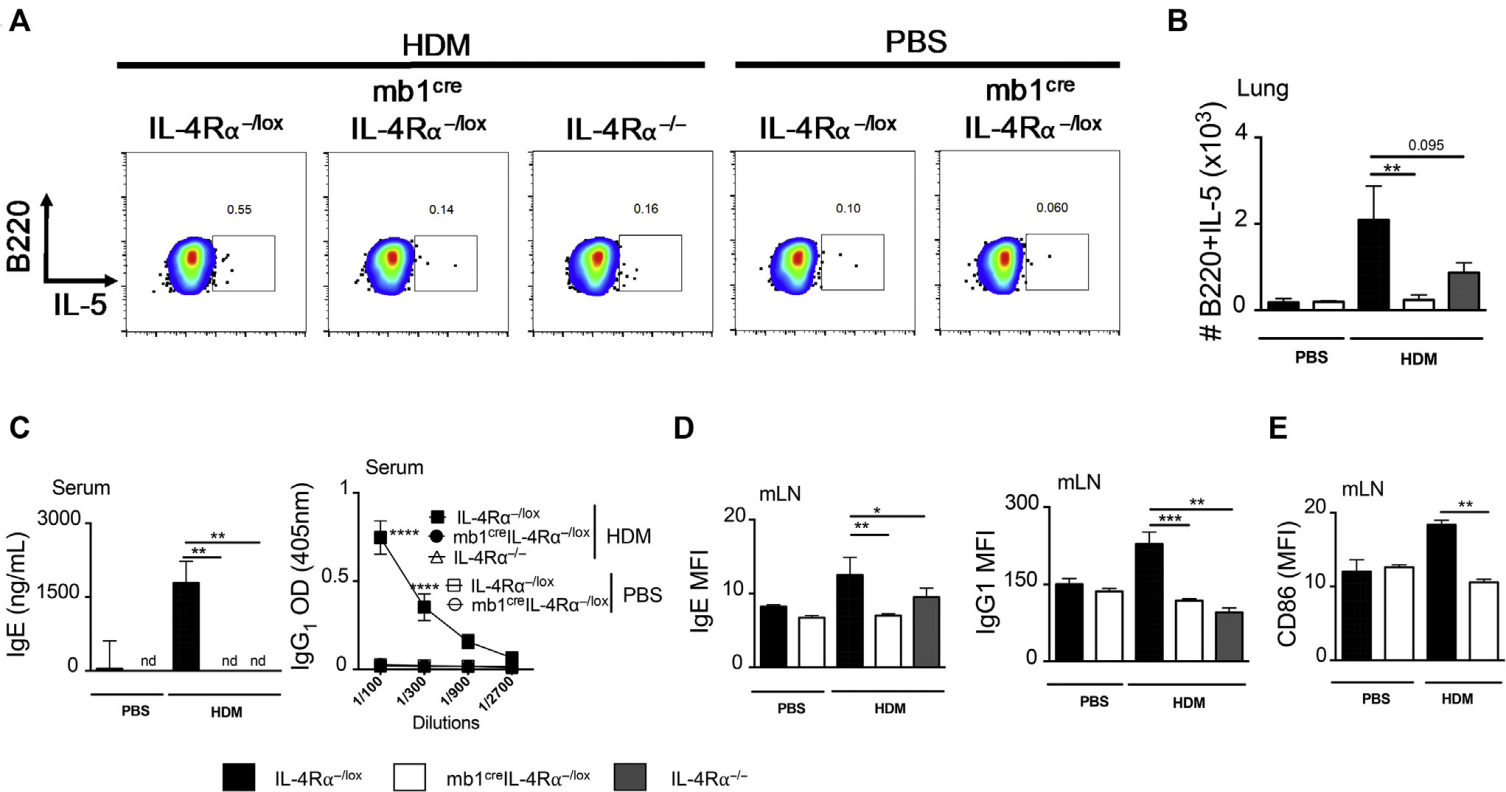
IL-4Rα signaling on B cells is essential for B effector 2 function and class switching. **A,** Representative flow cytometry plots of IL-5–producing B cells (live^+^B220^+^CD19^+^MHCII^+^IL-5^+^) in the lung of mb1^cre^IL-4Rα^−/lox^, littermate control IL-4Rα^−/lox^, and IL-4Rα^−/−^ mice. **B,** Quantification of total number of IL-5–producing B cells in the lung. **C,** Total serum IgE and HDM-specific IgG_1_ titers in mb1^cre^IL-4Rα^−/lox^, littermate control IL-4Rα^−/lox^, and IL-4Rα^−/−^ mice measured by ELISA. **D,** Surface expression of IgE (live^+^B220^+^CD19^+^MHCII^+^IgE^+^) and IgG_1_ (live^+^B220^+^CD19^+^MHCII^+^IgG1^+^) on mLN B cells, represented as MFI. **E,** CD86 surface expression on mLN B cells (Live^+^B220^+^CD19^+^MHCII^+^CD86^+^), represented as MFI. *MFI*, Median fluorescent intensity. Shown are means ± SDs from 1 representative experiment of 3 (n = 4-6). Significant differences between groups were analyzed by Student *t* test (Mann-Whitney) (Fig 4, *B*, *D*, and *E*) or by 2-way ANOVA with Benforroni posttest (Fig 4, *E*) and are described as ∗∗∗*P* < .001, ∗∗∗∗*P* < .0001.

**Fig E5:**
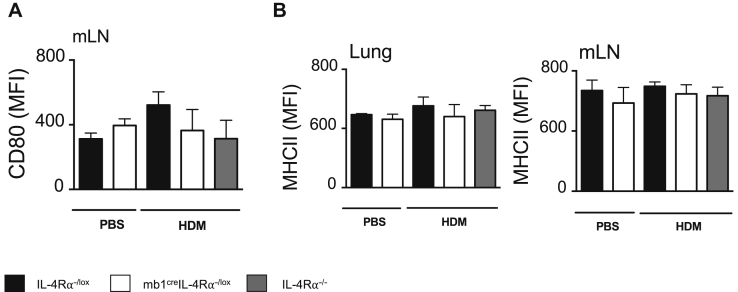
Antigen uptake and processing is intact in IL-4Rα–deficient B cells. **A,** Quantification of CD80 expression on B cells (live^+^B220^+^CD19^+^) in mLNs represented as MFI. **B,** Quantification of MHCII expression on B cells (live^+^B220^+^CD19^+^) in mLNs and lungs represented as MFI. *MFI*, Median fluorescent intensity. Shown are means ± SDs from 1 representative experiment of 3 (n = 6-7). Significant differences between groups were analyzed by Student *t* test (Mann-Whitney) and are described as ∗∗*P* < .01.

Effector B cells producing type 2 cytokines (B effector 2 [Be2]) have been shown to be important during parasitic infections, and early expression of IL-4 by these B cells promotes differentiation of type 2 CD4 T cells.^28^^,^^30^^,^^38^ We measured cytokine production by effector B cells and found increased frequencies and numbers of mLN B cells producing IL-5 in IL-4Rα^−/lox^ littermate mice when compared with mb1^cre^IL-4Rα^−/lox^ and IL-4Rα^−/−^ mice challenged with low-dose HDM (Fig 4, *A* and *B*, and Fig E4, *C*). We also observed a similar reduction in the number of IL-13–producing B cells in mb1^cre^IL-4Rα^−/lox^ and IL-4Rα^−/−^ when compared with IL-4Rα^−/lox^ littermate mice challenged with low-dose HDM (Fig E4, *D*). We then measured total serum IgE and HDM-specific IgG_1_ by ELISA and found significantly reduced titers in mb1^cre^IL-4Rα^−/lox^ and IL-4Rα^−/−^ when compared with IL-4Rα^−/lox^ littermate mice challenged with low-dose HDM (Fig 4, *C*). We analyzed IgE and IgG_1_ surface expression by B cells using flow cytometry. We found significantly reduced levels of IgE and IgG_1_ expression in B cells when comparing mb1^cre^IL-4Rα^−/lox^ to IL-4Rα^−/lox^ littermate mice (Fig 4, *D*). B- and T-cell engagement through CD86 and CD28 in T-cell zones is essential for T_FH_-cellgeneration and class switching to IgE.^39^ We measured CD86 and other costimulatory molecules on the surface of B cells and found reduced expression of CD86, but not CD80 and MHCII (Fig 4, *E*; see Fig E5, *A* and *B*, in this article’s Online Repository at www.jacionline.org), which may suggest an incomplete T-cell engagement via CD28 and explain lack of class switching. Thus far, our data suggested that IL-4Rα signaling on B cells is essential for Be2-cell function and class switching to IgE and this contributes to overall T_H_2 responses.

### IL-4Rα–responsive B cells are essential in type 2 airway inflammation during the effector phase

**Fig 5:**
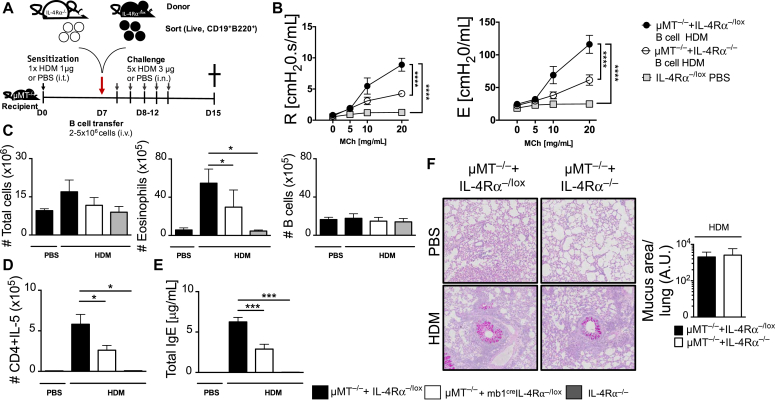
IL-4Rα signaling on B cells is essential at the effector phase of allergic asthma through regulation of AHR and T_H_2 airway responses. **A**, Schematic diagram showing sensitization and challenge protocol where μMT^−/−^ mice were sensitized with HDM 1 μg intratracheally on day 0 and naive B cells (live^+^B220^+^CD19^+^) from mb1^cre^IL-4Rα^−/lox^ or IL-4Rα^−/lox^ mice (2-5 × 10^6^ cells) were adoptively transferred intravenously a day before challenge with HDM 3 μg on days 8 to 12. Analysis was done on day 15. **B,** Airway resistance and elastance were measured with increasing doses of acetyl methacholine (0-20 mg/mL). **C,** Total lung cell numbers, eosinophil numbers, and B-cell numbers were stained and analyzed by flow cytometry and enumerated from % of live cells. **D,** Number of lung CD4 T cells producing IL-5 after 5-hour stimulation with PMA/ionomycin in the presence of monensin. **E,** Total serum IgE production from 2 independent experiments pooled together. **F,** Histology analyses of lung sections (magnification ×20), stained with periodic acid-Schiff. *A.U.*, Arbitrary units; *i.n.*, intranasal; *i.t.*, intratrachael; *i.v.*, intravenous; *PMA*, phorbal 12-myristate 13-acetate. Shown are means ± SEM from 2 independent experiments pooled (n = 10-14). Significant differences between groups were analyzed by Student *t* test (Mann-Whitney) (Fig 5, *C-F*) by 2-way ANOVA with Benforroni posttest (Fig 5, *B*) and are described as ∗*P* < .05, ∗∗∗*P* < .001, ∗∗∗∗*P* < .0001.

Previous studies had suggested that B cells were important in T_FH_-cell development at the sensitization stage, but not at the effector stage, and played minimal role in disease if adoptively transferred after sensitization.^13^ We asked whether IL-4Rα–responsive B cells were important only at the sensitization stage. We sensitized B-cell–deficient μMT^−/−^ mice with low-dose HDM and a day before challenge, we transferred naive B cells having either sufficient or lacking IL-4Rα (Fig 5, *A*). We found that μMT^−/−^ with B cells from mb1^cre^IL-4Rα^−/lox^ mice had significantly reduced resistance and elastance when compared with μMT^−/−^ receiving B cells from IL-4Rα^−/lox^ littermate mice (Fig 5, *B*). This reduced AHR in mb1^cre^IL-4Rα^−/lox^ mice was accompanied by reduced eosinophil recruitment in the lung, but not total cell or B-cell numbers (Fig 5, *C*). We then analyzed CD4 T-cell numbers that were producing type 2 cytokines and found significantly reduced IL-5– producing CD4 T cells in the lung of μMT^−/−^ mice that received mb1^cre^IL-4Rα^−/lox^ B cells when compared with μMT^−/−^ mice receiving IL-4Rα^−/lox^ B cells (Fig 5, *D*). We then measured total IgE by ELISA and found significantly increased IgE in μMT^−/−^ mice that received IL-4Rα^−/lox^ B cells compared with μMT^−/−^ mice that received mb1^cre^IL-4Rα^−/lox^ B cells (Fig 5, *E*). We observed comparable mucus area between μMT^−/−^ mice receiving IL-4Rα^−/lox^ B cells or mb1^cre^IL-4Rα^−/lox^ B cells (Fig 5, *F*). No mucus-producing cells were detected in control mice challenged with saline (Fig 5, *F*). Our findings suggested that IL-4Rα signaling on B cells was also essential at the effector phase for optimal T_H_2 airway responses.

### IL-4/IL-13–producing B cells contribute to AHR but not inflammation

**Fig 6:**
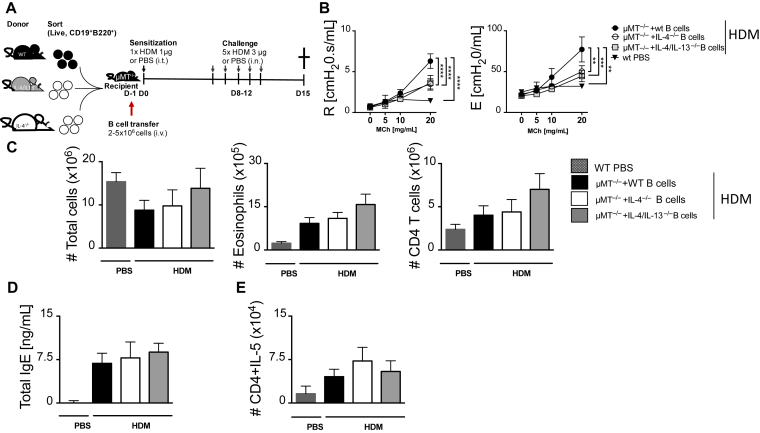
T_H_2 cytokine production by B cells is important only in regulation of AHR but not eosinophilia or T_H_2 airway responses. **A**, Schematic diagram showing sensitization and challenge protocol where naive B cells (live^+^B220^+^CD19^+^) from IL-4^−/−^ or IL-4^−/−^IL-13^−/−^ mice (2-5 × 10^6^ cells) were adoptively transferred intravenously into μMT^−/−^ mice a day before sensitization with HDM 1 μg intratracheally on day 0 and challenged with HDM 3 μg on days 8 to 12. Analysis was done on day 15. **B,** Airway resistance and elastance were measured with increasing doses of acetyl methacholine (0-20 mg/mL). **C,** Total lung cell numbers, eosinophil numbers, and CD4 T-cell numbers were stained and analyzed by flow cytometry and enumerated from % of live cells. **D,** Total IgE production from 2 independent experiments pooled together. **E,** Number of lung CD4 T cells producing IL-5 after 5-hour stimulation with PMA/ionomycin in the presence of monensin. *i.n.*, Intranasal; *i.t.*, intratrachael; *i.v.*, intravenous; *PMA*, phorbal 12-myristate 13-acetate.Shown are means ± SEM from 2 independent experiments pooled (n = 10-14). Significant differences between groups were analyzed by Student *t* test (Mann-Whitney) (Fig 6, *C-F*) by 2-way ANOVA with Benforroni posttest (Fig 6, *B*) and are described as ∗*P* < .05, ∗∗∗*P* < .001, ∗∗∗∗*P* < .0001.

We then investigated whether the production of type 2 cytokines by these B cells is essential for allergic airway inflammation. We adoptively transferred naive B cells either sufficient or deficient of IL-4 or double deficient of IL-4/IL-13 into μMT^−/−^ mice, sensitized and challenged with low-dose HDM (Fig 6, *A*). We measured lung function and found both resistance and elastance to be significantly reduced in μMT^−/−^ mice that received IL-4–deficient or IL-4/IL-13–double-deficient B cells when compared with μMT^−/−^ mice that received wild-type B cells (Fig 6, *B*). We then measured cellular infiltrate in the lung and found no major changes in total lung infiltrate, eosinophils, and CD4 T cells in all recipient mice (Fig 6, *C*). The number of B cells was lower in μMT^−/−^ mice that were adoptively transferred with B cells from various strains compared with control mice; however, no differences were observed between mice that were challenged with a low dose of HDM (Fig 6, *C*). Total IgE measured by ELISA was not changed between groups of μMT^−/−^ mice adoptively transferred with B cells and sensitized and challenged with low-dose HDM (Fig 6, *D*). We also did not observe any changes in type 2 cytokines produced by CD4 T cells in the absence of type 2 cytokines produced by B cells when comparing μMT^−/−^ mice that received wild-type B cells to those that received IL-4–deficient or IL-4/IL-13–double-deficient B cells (Fig 6, *E*). Taken together, these results suggested that although B cells producing type 2 cytokines are essential in AHR, they play minimal role in airway inflammation.

## Discussion

B cells secrete IgE and are important in activating mast cells and basophils degranulation, which initiates a cascade of inflammatory signals. However, contradictory findings on the requirement of B cells exist in studies using mice lacking B cells. More recent evidence suggested that antigen load determines the importance of B cells, particularly in interactions with T_H_ cells and generation of T_FH_ cells. IL-4 is critical in class switching of B cells to generate IgE^40^; however, whether signaling through the IL-4Rα on B cells is required for the generation of T_FH_ cells and IgE has not been investigated in the context of allergic asthma. Here, we showed that IL-4Rα–responsive B cells are important mainly when the load of HDM antigen is limiting. We further showed that IL-4Rα–responsive B cells regulate AHR, IgE secretion, and T_FH_-cell generation, and that B-cell–derived type 2 cytokines are required for optimal T_H_2 responses.

We first challenged mice with high-dose HDM and found IL-4Rα–responsive B cells to be important in AHR and IgE production, but not for eosinophil recruitment or type 2 cytokine production. This is consistent with previous studies where B-cell–deficient mice showed similar levels of eosinophilia and type 2 cytokines when sensitized and challenged with high-dose HDM.^14^ B-cell–deficient mice have significantly increased AHR at high-dose HDM, a phenotype different to what we observe in IL-4Rα–deficient B cells, which suggested that IL-4Rα–responsive B cells may be involved in driving AHR during allergic asthma. Differences in susceptibility to disease is also observed between μMT^−/−^ mice and mb1^cre^IL-4Rα^−/lox^ mice during chronic *Schistosoma mansoni* infection, which is attributed to differences in immunomodulatory IL-10 production seen in μMT^−/−^ mice, but not in mb1^cre^IL-4Rα^−/lox^ mice.^28^^,^^29^

Similar to what was reported by Dullaers et al,^14^ mb1^cre^IL-4Rα^−/lox^ mice that were sensitized with 1 μg HDM and challenged with 3 μg HDM displayed a great reduction in AHR, eosinophil recruitment, and type 2 cytokine production compared with littermate IL-4Rα^−/lox^ mice. We also observed significant reduction in GC B cells and T_FH_ cells, but no changes in follicular and slight increase in marginal-zone B cells, probably as a compensatory mechanism to increase non-GC antibody production. The ability of B cells to produce type 2 cytokines was also significantly reduced, suggesting that IL-4Rα B cells help contribute to the overall type 2 immune response output. This is consistent with previous studies, where IL-4Rα responsiveness by B cells is crucial in early IL-4 production in mLNs, defining a dichotomy in subsequent CD4 T_H_-cell differentiation.^28^, ^29^, ^30^^,^^38^ B cells receive the IL-4 signal from CD4 T cells in GCs to initiate class switching to produce IgE, which is likely to be dependent on IL-4Rα signaling on B cells.^33^^,^^41^ Although IgE can directly be generated from IgM, particularly with low antigen load,^42^ we think that in our model, lack of IL-4Rα signaling on B cells led to reduced sequential class switching from IgM to IgG_1_ and to IgE. Sequential class switching occurs in the GCs and results in high-affinity plasma cell IgE.^41^^,^^43^^,^^44^ These high-affinity IgE plasma cells are direct precursors of IgG_1_ plasma cells because they share similar CDR3 repertoire in the context of helminth infections, skin cancers, and alum/ovalbumin-induced asthma.^44^^,^^45^ In human B cells from the tonsil, IL-4Rα signaling is required for GC maintenance and generation of high-affinity IgE BCRs that select for plasma cell compartments.^46^^,^^47^

There has been contrasting evidence regarding whether B cells are important during the sensitization phase, the effector phase, or in both phases of allergic response.^13^^,^^15^ B cells were found to be critical in shaping IL-4–committed T_FH_ cells during HDM sensitization stage, which contributed to the effector T_H_2 pool during challenge stages. However, blocking of T_FH_ cells with BCL6 inhibitor after the sensitization stage did not reduce T_H_2 allergic airway inflammation, which was attributed to the redundant role of B cells at this stage. This is in contrast with recent findings where blocking B cells with anti-CD20 before HDM challenge significantly reduced T_H_2 airway responses.^15^ To consolidate these findings, we transferred IL-4Rα–deficient B cells after HDM sensitization and before challenge. Our data showed that IL-4Rα–responsive B cells are required at the challenge stage, because we observed reduced AHR, T_H_2 cytokines, and total IgE when we transferred B cells lacking IL-4Rα into recipient mice. Both previous studies had used a similar dose of 20 μg of HDM to sensitize and challenge and we used 3 μg of HDM to challenge. It is likely that the choice of method used to target B cells or their function might be a major contributing factor between the 2 studies and not necessarily the load of HDM antigen. In our studies, we transferred naive B cells a day before challenge, whereas Ballesteros-Tato et al^13^ had blocked T_FH_ cells using BCL6 inhibitor and Wypych et al^15^ targeted B cells using anti-CD20 mAb. All in all, our data demonstrated that IL-4Rα on B cells is important in T_H_2 allergic asthma at both sensitization and challenge stages and contributes to overall T_H_2 responses when the antigen load is limited.

Previous studies have shown a controversial role of IL-21 in T_FH_ cells that eventually developed into committed T_H_2 cells. Ballesteros-Tato et al^13^ suggested that lung T_H_2 cells were direct descendants of IL-21^+^Bcl6^+^ T_FH_ cells and developed 6 days after multiple sensitization with 25 μg of HDM exposure. In contrast, Coquet et al^37^ found that IL-21^+^ T_FH_ cells did not differentiate efficiently into ST2^+^ T_H_2 cells and migrated into the lung without all key features of T_FH_ cells such as CXCR5 expression. This idea was recently supported by Tibbitt et al,^48^ where a trajectory single-cell analysis of differentiating T_H_2 cells up until day 10 suggested that naive CD4 T cells acquired many features of T_FH_ cells but did not express Bcl6 or CXCR5, which suggested that T_H_2 cells did not descend directly from GC T_FH_-cell precursors. In our study, the absence of IL-4Rα signaling on B cells resulted in reduced IL-21 production in the lung, which might explain reduced GCs and T_H_2 cells. Appropriate experiments to answer this complex function of IL-21 in TFH cells that commit to T_H_2 cells are needed and should use a double (Bcl6 and IL-21) or triple (Bcl6, IL-21 and IL-4) reporter transgenic mouse or a fate reporter transgenic mouse that can trace naive CD4 T cells as they differentiate into intermediate and committed T_H_2 cells in multiple tissues.

Be2 cells producing IL-4 or IL-13 have been shown to be important in worm expulsion or in *Leshmania major* disease susceptibility.^28^, ^29^, ^30^^,^^38^^,^^49^ These Be2 cells are dependent on IL-4 and IL-4Rα and require the presence of intact T_H_2 cells.^28^^,^^38^^,^^49^ Because we had observed that IL-4Rα signaling on B cells was essential for optimal T_H_2 allergic airway immune responses, we then investigated whether production of cytokines by these Be2 cells was essential for optimal T_H_2 immune responses. We transferred B cells from IL-4 or IL-4/IL-13–deficient mice into μMT^−/−^ before sensitization. Interestingly, Be2 cells were essential for AHR, but played no role in lung eosinophil recruitment, total IgE production, or type 2 cytokine production by CD4 T cells. This suggested that although the presence of IL-4/IL-13 cytokine production by Be2 cells was required for AHR, it was redundant in other parameters. It is likely that T_H_2 cells can compensate for the lack of IL-4/IL-13 production by B cells; however, how T_H_2 cells fail to compensate for AHR is currently unclear and requires further investigation. B cells in lymph nodes secrete early IL-4 production, which may be important for CD4 T-cell differentiation.^28^^,^^29^ It is likely that B cells produce early IL-4 in mLNs, which act in an autocrine fashion to upregulate IL-4Rα, but whether this IL-4 plays a major role in CD4 T_H_2 differentiation, we can only speculate.

Blocking B cells with anti-CD20 before sensitization did not affect T_H_2 cytokine production *ex vivo*, but resulted in reduction in eosinophils and IFN-γ secretion.^15^ Unfortunately, AHR was not investigated in this setting, making it difficult to draw parallel conclusions regarding the function of B cells in AHR. We can speculate that other intrinsic Be2-cell mechanisms are at play in regulation AHR. B cells are known to take up HDM and present it to naive T cells, priming them to become T_H_2 cells both *in vitro* and *in vivo*, and lack of MHCII in B cells results in reduced T_H_2 priming.^14^^,^^15^ IL-4Rα–deficient B cells have been shown to have reduced MHC II expression and antigen uptake, which contributed in reduced T_H_2 priming on secondary exposure to *Nippostrongylus brasiliensis*, leading to increased worm burdens.^30^ We did not observe any changes in CD80 or MHCII expression on IL-4Rα signaling–deficient B cells when compared with IL-4Rα–sufficient mice (Fig E5, *A* and *B*). However, we did observe a reduction in CD86 costimulatory molecule (Fig 4, *E*), which may suggest an incomplete T-cell engagement via CD28 and reduced IgE potentiation.^39^ Our findings do not suggest antigen uptake and processing as a potential mechanism for reduced T_H_2 priming, but a lack of complete costimulatory engagement in the absence of IL-4Rα signaling on B cells.

### Conclusions

We showed that IL-4Rα–responsive B cells play a nonredundant role in allergic asthma in an antigen load–dependent manner. We further showed that IL-4Rα signaling on B cells is crucial at both sensitization and challenge stages and produces cytokines that help in optimal T_H_2 allergic airway responses. We further showed that Be2-cell function is important only for AHR but redundant in eosinophilia. Our study highlighted a previously unappreciated function of IL-4Rα signaling on B cells and brings evidence for targeting of this signaling axis in allergic asthma.

**Key messages**

• IL-4R**α**–responsive B cells play a critical role in HDM-induced allergic asthma when the load of HDM is limited.
• IL-4R**α** signaling on B cells is required at both sensitization and effector stages of allergic disease.
• IL-4R**α**–responsive B cells are required for Be2 function of B cells and help maintain optimal T_H_2 during allergic asthma.
